# Amino acid racemization reveals differential protein turnover in osteoarthritic articular and meniscal cartilages

**DOI:** 10.1186/ar2639

**Published:** 2009-03-06

**Authors:** Thomas V Stabler, Samuel S Byers, Robert D Zura, Virginia Byers Kraus

**Affiliations:** 1 Department of Medicine, Duke University Medical Center, Box 3416, Durham, NC 27710, USA; 2 Department of Dentistry, Case Western Reserve University, 10900 Euclid Avenue, Cleveland, OH 44104, USA; 3 Department of Surgery, Duke University Medical Center, Box 3205, Durham, NC 27710, USA

## Abstract

**Introduction:**

Certain amino acids within proteins have been reported to change from the L form to the D form over time. This process is known as racemization and is most likely to occur in long-lived low-turnover tissues such as normal cartilage. We hypothesized that diseased tissue, as found in an osteoarthritic (OA) joint, would have increased turnover reflected by a decrease in the racemized amino acid content.

**Methods:**

Using high-performance liquid chromatography methods, we quantified the L and D forms of amino acids reported to racemize *in vivo *on a biological timescale: alanine, aspartate (Asp), asparagine (Asn), glutamate, glutamine, isoleucine, leucine (Leu), and serine (Ser). Furthermore, using a metabolically inactive control material (tooth dentin) and a control material with normal metabolism (normal articular cartilage), we developed an age adjustment in order to make inferences about the state of protein turnover in cartilage and meniscus.

**Results:**

In the metabolically inactive control material (n = 25, ages 13 to 80 years) and the normal metabolizing control material (n = 19, ages 17 to 83 years), only Asp + Asn (Asx), Ser, and Leu showed a significant change (increase) in racemization with age (*P *< 0.01). The age-adjusted proportions of racemized to total amino acid (D/D+L expressed as a percentage of the control material) for Asx, Ser, and Leu when compared with the normal articular cartilage control were 97%, 74%, and 73% in OA meniscal cartilage and 97%, 70%, and 78% in OA articular cartilage. We also observed lower amino acid content in OA articular and meniscal cartilages compared with normal articular cartilage as well as a loss of total amino acids with age in the OA meniscal but not the OA articular cartilage.

**Conclusions:**

These data demonstrate comparable anabolic responses for non-lesioned OA articular cartilage and OA meniscal cartilage but an excess of catabolism over anabolism for the meniscal cartilage.

## Introduction

Amino acids, with the exception of glycine, can occur in two stereoisomeric forms: D and L. However, only the L forms are incorporated into mammalian proteins. Certain L amino acids within proteins are prone to racemization over time [1]. In proteins with low turnover, this racemization leads to an age-dependent increase of D amino acids. Racemization has been observed in a variety of human tissues, including cartilage, lens, brain, lung, aorta, skin, tooth, and bone [2-10]. In non-metabolizing tissues, racemization, as reflected by the D/D+L aspartate (Asp) ratio, increases linearly with age. All methods of D and L Asp measurement involve high-temperature acid hydrolysis that induces a certain time- and temperature-dependent background racemization; this hampers the ready comparison of results across studies. However, successful management of background variability is possible by employing a precise experimental protocol for all samples.

Tissue catabolism alone leads to no apparent change in the rate of D amino acid accumulation and would be manifested as a linear increase of tissue D/D+L amino acid content per unit of tissue mass over time with age. This would be analogous to cutting off a wedge of cheese from a large wheel, which would not affect the properties of the remaining portion. Alternatively, an increase in tissue anabolism alone should lead to no change in the age-related rate of D amino acid accumulation but a reduction in the rate of D/D+L amino acid accumulation per unit of tissue mass due to additions of newly synthesized L amino acids. The combination of catabolism and anabolism could lead to the replacement of D amino acids for L amino acids, resulting in a reduction of the rate of D/D+L amino acid accumulation. Thus, a study of the changes in protein aging within a tissue, reflected by racemization rates and quantities of racemized material, has the potential to yield valuable insights into tissue turnover and the condition of molecules that are being released into the general circulation as biomarkers of a given disease process. Moreover, the quantification of 'aged' circulating biochemical markers, which we call 'biomarker dating', might further improve their predictive capabilities for pathological tissue turnover states.

The purpose of this study was to examine protein turnover of osteoarthritic (OA) articular hyaline cartilage and meniscal fibrocartilage compared with normal articular hyaline cartilage through the analysis of racemization of Asp, asparagine (Asn), glutamate (Glu), glutamine (Gln), serine (Ser), alanine (Ala), leucine (Leu), and isoleucine (Ile). To validate our method, we also examined racemization of these amino acids in human dentin, a tissue shown by radiochemical methodology to be metabolically inactive and hence not subject to protein turnover [11]. The control materials (normal articular hyaline cartilage and tooth dentin) provided benchmarks for comparison of racemization rates since they had the same inherent background. Moreover, we have innovated the application of age-adjusted racemization data, based upon control values, to compare metabolism rates across tissues and amino acids. These data further underscored the benefit and utility of including a range of age-matched non-metabolizing controls in any study of the racemization of metabolizing tissue.

## Materials and methods

### Tissue procurement

According to the institutional review boards at Duke University Medical Center (Durham, NC, USA) and Case Western Reserve University (Cleveland, OH, USA), our protocol for using anonymous waste tissue met the definition of research not involving human subjects and satisfied the Privacy Rule.

### Osteoarthritic knee joint tissue preparation

Surgical waste tissues were obtained from 30 osteoarthritis patients who were undergoing total knee joint replacement at Duke University Medical Center. Ages ranged from 43 to 85 years, and there were 17 females and 13 males. Articular and meniscal cartilages were isolated from sections of the joint which showed no apparent macroscopic damage. Tissues were frozen at -80°C until analysis.

### Normal cartilage tissue preparation

Surgical waste tissues from acute joint trauma and human cadaveric normal articular cartilages were obtained from 19 subjects through Duke University Medical Center and the National Disease Research Interchange (Philadelphia, PA, USA). Ages ranged from 17 to 83 years, and there were 13 males and 6 females. Normal cartilage was defined as cartilage from subjects with no joint disease of any kind. Tissues were frozen at -80°C until analysis.

### Tooth root dentin preparation

Non-deciduous teeth (molars) were obtained as surgical waste tissue from 25 individuals (13 females and 12 males) undergoing tooth extraction, with ages ranging from 13 to 80 years. The roots were separated from the crown, cleaned, sterilized with bleach, and then demineralized at room temperature in a solution of 0.5 M EDTA (ethylenediaminetetraacetic acid) for approximately 3 months with one change of solution during that time. Root dentin was dissected out from the demineralized tooth, and great care was taken to exclude pulp and enamel.

### Sample hydrolysis

The articular and meniscal cartilages and dentin samples were first pulverized under liquid nitrogen using a Bio-Pulverizer (BioSpec Products Inc., Bartlesville, OK, USA), followed by homogenization in cold 6 M HCl for 3 minutes using a Mini-Beadbeater-8 (BioSpec Products Inc.). Samples were immediately transferred to glass tubes and adjusted to a final concentration of 20 mg of tissue per milliliter of 6 M HCl. All proteins in the samples were hydrolyzed into their individual amino acids by heating for 8 hours at 105°C, followed by rapid neutralization on ice with 6 N NaOH. The hydrolyzed samples were stored at -80°C until analysis. We found in preliminary studies (data not shown) that the acid hydrolysis procedure induced racemization and that the rate of this methodologically induced racemization was dependent on not only time and temperature, but also the amount of tissue. Rather than use a correction factor for this artifactual racemization, we chose to exercise great care in treating all samples identically in regard to time, temperature, and amount of tissue used and to report the values without correction.

### Amino acid derivatization

The amino acids within the hydrolyzed samples were derivatized using a previously described method [12]. Specifically, 10 μL of the neutralized sample hydrolysate, to which 10 μL of an internal standard had been added, was buffered with 155 μL of 0.4 M boric acid (pH 9.0), followed by the addition of 25 μL of derivatization reagent. The derivatization reagent consisted of 20 mg/mL each of o-phthaldialdehyde and *N*-tertiary-butyloxycarbonyl-L-cysteine, both purchased from Sigma-Aldrich (St. Louis, MO, USA) and made fresh daily in methanol. Samples were mixed, and after a 1-minute incubation, the resulting fluorescent diasterioisomeric isoindolyl derivatives were separated and quantified using reversed-phase high-performance liquid chromatography (HPLC).

### High-performance liquid chromatography analysis

We used the HPLC method of Hashimoto and colleagues [12] with modifications. The HPLC system consisted of an HP1090 II liquid chromatograph (Agilent Technologies Inc., Santa Clara, CA, USA) and a Jasco FP-1520 fluorescence detector (Jasco Inc., Easton, MD, USA) set to an excitation of 344 nM and an emission of 443 nM. A Chromolith RP-18e 100 × 4.6 mm column (VWR International LLC, West Chester, PA, USA) was used for the separation with an injection volume of 20 μL and a constant flow rate of 1 mL/minute. Two injections were required per sample with different mobile phases and gradients. The separation of D and L forms of Asp, Glu, Ser, and Ala was accomplished using a mobile phase consisting of 0.2 M acetic acid adjusted to a pH of 6.0 with NaOH and acetonitrile. The acetonitrile gradient was as follows: initially 8%, increasing 0.2%/minute for the first 30 minutes, 0.33%/minute for the next 18 minutes, and 0.66%/minute for the final 12 minutes for a concentration of acetonitrile of 28% at 60 minutes. All mobile phases were degassed using helium sparging. We found that the hydrolysis procedure converted all D and L Asn and Gln to D and L Asp and Glu. All results for Asp and Glu are therefore measurements of Asp + Asn (Asx) and Glu + Gln (Glx).

The separation of D and L forms of Leu and Ile required substitution of methanol for the acetonitrile and a methanol gradient as follows: initially 35%, increasing 0.454%/minute for the first 55 minutes and then 3.33%/minute for the next 3 minutes to a final concentration of methanol of 70% and holding at that concentration for an additional 2 minutes. The time from derivatization of sample to injection was kept constant at 5 minutes for all samples.

Several different concentrations of each individual D and L amino acid standard (Sigma-Aldrich) were run, and the resulting peak areas were used to construct calibration curves using a linear regression model. These calibration curves were then used to quantify the unknown samples.

The detection limit for this method, as defined by a peak height of twice the baseline noise, was determined to be 0.005 nmol/mg for all D and L amino acids. The lowest level of D or L amino acid detected within any sample (tooth, cartilage, or meniscus) was 0.03 nmol/mg.

### Statistical analysis

Analyses were performed using GraphPad Prism4 software (GraphPad Software Inc., San Diego, CA, USA). Linear regression was used to compare amino acid concentrations and D/D+L ratios versus age. Age-adjusted proportions of racemized amino acids were calculated based on the dentin control material as well as the normal articular cartilage material in order to evaluate the relative turnover of articular cartilage versus individually matched meniscal cartilages, assessed by paired *t *test, and to evaluate the relative turnover of the various amino acids, assessed by analysis of variance (ANOVA). A *P *value of less than 0.05 was considered statistically significant.

## Results

Analyses of dentin from non-deciduous teeth were performed to determine the maximal rates of racemization with biological aging for Asx, Glx, Ser, Ala, Leu, and Ile and thereby to determine the amino acids that might be of value for biomarker-dating purposes. We found the relative mean ratios of D to total (D+L) amino acids in our control material (dentin) to be Asx > Ser > Ala ≈ Glx > Leu > Ile (Table 1) (age-related rates of accumulation by slope were Asx > Ser > Leu > Ala ≈ Glx > Ile). However, only Asx, Ser, and Leu showed a significant increase with age, thereby validating the analysis of these three amino acids for the purposes of evaluating tissue turnover. Just as in the dentin control, only Asx, Ser, and Leu showed a significant increase with age in normal articular cartilage and with the same rank order of age-related accumulation as for dentin (Asx > Ser > Leu).

**Table 1:** Association of D/D+L amino acid ratios with age in tooth dentin, normal cartilage, and paired osteoarthritic articular and meniscal cartilages

		Amino acid					
		
		Asx	Ser	Leu	Ile	Glx	Ala
Tooth dentin	Mean D/D+L ratio	0.0599	0.0198	0.0105	0.0077	0.0137	0.0135
	*r*^2^	0.961	0.827	0.349	0.018	0.014	0.002
	Slope e-10^5^	65.2	30.5	6.9	-3.4	0.5	0.6
	*P *value	<0.0001^a^	<0.0001^a^	0.0019^a^	0.5246	0.5740	0.8497

Normal articular cartilage	Mean D/D+L ratio	0.0463	0.0142	0.0117	0.0125	0.0125	0.0283
	*r*^2^	0.778	0.470	0.287	0.060	0.194	0.001
	Slope e-10^5^	35.2	18.4	11.6	0.7	2.3	-0.6
	*P *value	<0.0001^a^	0.0012^a^	0.0181^a^	0.3136	0.0590	0.9410

OA articular cartilage	Mean D/D+L ratio	0.0506	0.0120	0.0105			
	*r*^2^	0.126	0.173	0.053			
	Slope e-10^5^	16.0	7.0	6.0			
	*P *value	0.081	0.055	0.221			
			
OA meniscal cartilage	Mean D/D+L ratio	0.0506	0.0127	0.0099			
	*r*^2^	0.021	0.002	0.0099			
	Slope e-10^5^	8.0	1.0	-3.0			
	*P *value	0.442	0.802	0.595			

Dentin: n = 25 human tooth samples from subjects ranging in age from 13 to 80 years. Normal cartilage: n = 19 human surgical waste and autopsy samples from subjects ranging in age from 17 to 83 years. Osteoarthritic (OA) articular and meniscal cartilages: n = 30 paired human surgical waste samples from subjects ranging in age from 43 to 85 years. ^a^Denotes significant age-related increase at *P *< 0.05. Ala, alanine; Asx, asparagine + aspartate; Glx, glutamate + glutamine; Ile, isoleucine; Leu, leucine; Ser, serine.

**Figure 1:**
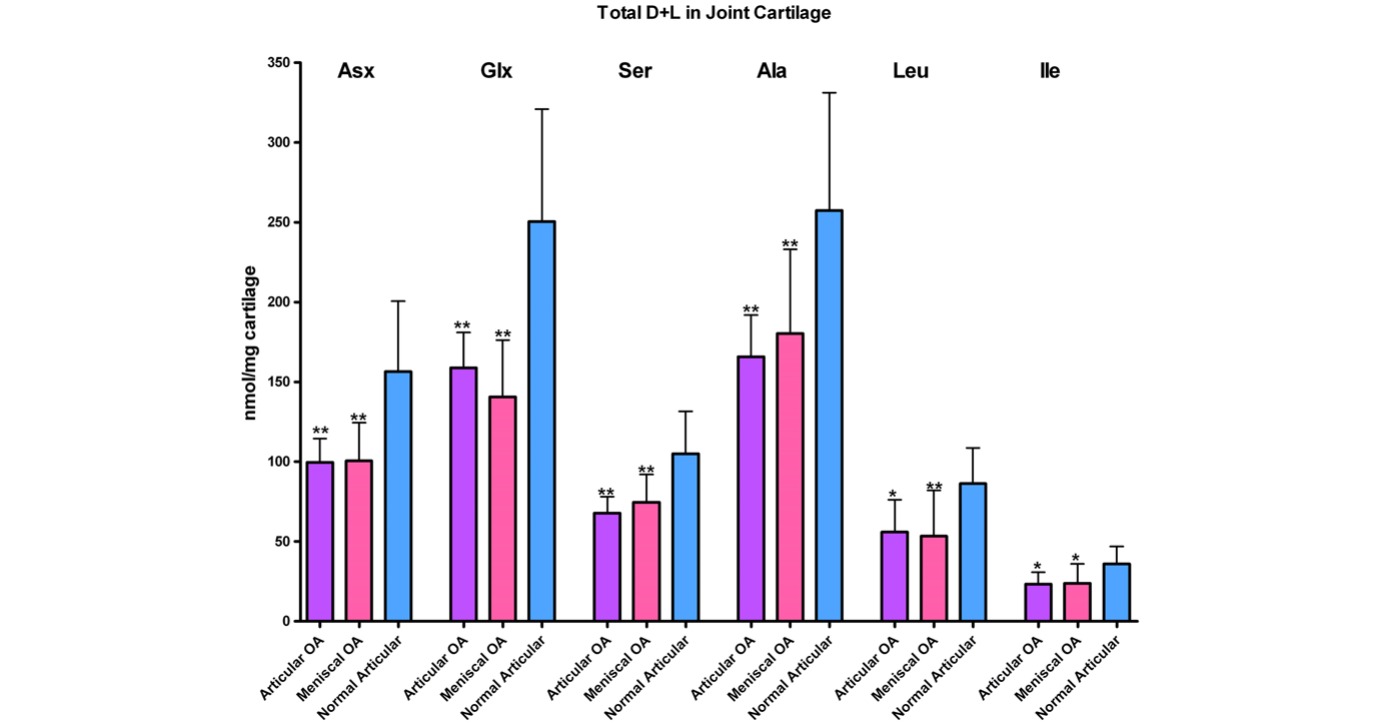
Differences in mean total amino acids D+L (nmol/mg + standard deviation) between normal articular cartilage (n = 12 age-matched samples), paired osteoarthritic (OA) articular cartilages (n = 30), and OA meniscal cartilages (n = 30). The normal tissue was age-matched to the OA tissues. Analysis of variance results were *P *< 0.0001 for asparagine + aspartate (Asx), glutamate + glutamine (Glx), serine (Ser), and alanine (Ala); *P *< 0.0005 for leucine (Leu); and *P *= 0.0012 for isoleucine (Ile). Significance of change from normal cartilage by Bonferroni post test was *P < 0.01 and **P < 0.001. Light blue represents normal articular cartilage, purple represents OA articular cartilage, and pink represents OA meniscal cartilage.

To measure the change in protein composition within articular and meniscal cartilages, we first analyzed total amino acid content (amount of D+L forms normalized to tissue wet weight) for all six amino acids. In meniscal cartilage, the concentrations of total Asx, Glx, Ser, Ala, Leu, and Ile all decreased significantly with age. The mean amino acid content did not vary significantly with age for normal or OA articular cartilage (Table 2). However, when the mean total amino acid contents between age-matched normal articular cartilage and OA articular and meniscal cartilages were compared, there was a significantly lower mean concentration of each of the amino acids in the OA cartilages (Figure 1).

**Table 2:** Change in total (L+D) amino acid composition with age in normal cartilage, osteoarthritic articular, and meniscal cartilages

		Amino acid					
		Asx	Ser	Leu	Ile	Glx	Ala
Normal articular cartilage	Mean (nmol/mg)	157.3	104.8	87.4	37.2	252.5	262
	*r*^2^	0.054	0.037	0.042	0.065	0.060	0.090
	Slope	-0.456	-0.233	-0.203	-0.120	-0.796	-1.040
	*P *value	0.327	0.418	0.399	0.293	0.297	0.198

OA articular cartilage	Mean (nmol/mg)	99.5	67.7	56.0	23.2	159.1	165.7
	*r*^2^	0.076	0.070	0.028	0.081	0.056	0.059
	Slope	0.419	0.278	0.237	0.196	0.532	0.645
	*P *value	0.141	0.157	0.377	0.129	0.208	0.196

OA meniscal cartilage	Mean (nmol/mg)	100.4	74.6	53.4	23.7	140.5	180.4
	*r*^2^	0.208	0.194	0.143	0.144	0.194	0.232
	Slope	-1.11	-0.78	-1.06	-0.458	-1.59	-2.57
	*P *value	0.011^a^	0.015^a^	0.040^a^	0.039^a^	0.015^a^	0.007^a^

Composition is presented in nanomoles per milligram of tissue wet weight. ^a^Denotes significant value. Ala, alanine; Asx, asparagine + aspartate; Glx, glutamate + glutamine; Ile, isoleucine; Leu, leucine; OA, osteoarthritic; Ser, serine.

We next analyzed the D/D+L ratios of only those amino acids that showed a significant change with age in the control materials (dentin and normal articular cartilage) (Table 1). Although the complements of tissue proteins in teeth are different from those in hyaline articular cartilage and meniscal fibrocartilage, it is nevertheless possible across tissues to compare rates of amino acid racemization as estimated by the D/D+L ratio of these amino acids in individuals of various ages. As for the dentin control material and normal articular cartilage, the OA cartilages (articular and meniscal) showed the same relative rates of age-related racemization as the control tissues (Asx > Ser > Leu). However, there was no significant age-related accumulation of racemized amino acids in the OA cartilages as there was for the normal cartilage.

**Figure 2:**
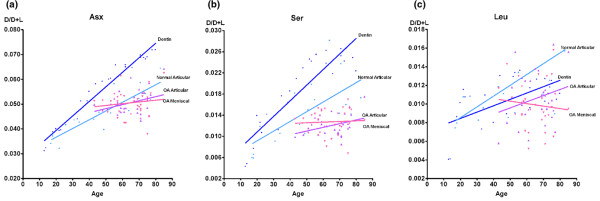
Amino acid ratio D/D+L versus age with selected regression lines from Table 1. **(a) **Asparagine + aspartate (Asx): dentin *r*^2 ^= 0.961, normal articular cartilage *r*^2 ^= 0.778, osteoarthritic (OA) articular cartilage *r*^2 ^= 0.126, and OA meniscal cartilage *r*^2 ^= 0.021. **(b) **Serine (Ser): dentin *r*^2 ^= 0.827, normal articular cartilage *r*^2 ^= 0.470, OA articular cartilage *r*^2 ^= 0.173, and OA meniscal cartilage *r*^2 ^= 0.127. **(c) **Leucine (Leu): dentin *r*^2 ^= 0.349, normal articular cartilage *r*^2 ^= 0.287, OA articular cartilage *r*^2 ^= 0.053, and OA meniscal cartilage *r*^2 ^= 0.010. The four tissues studied are indicated by regression line colors: dentin (dark blue), normal articular cartilage (light blue), OA articular cartilage (purple), and OA meniscal cartilage (pink).

The age-related racemization (linear regression slopes) of articular and meniscal cartilage extracellular matrix proteins (D/D+L ratio by age) was compared with the dentin standard. Compared with Asx in dentin, joint tissue Asx racemized at 54% (normal articular cartilage), 14% (OA meniscal cartilage), and 28% (OA articular cartilage) of the rate in dentin (Figure 2). Compared with Ser in dentin, joint tissue Ser racemized at 60% (normal articular cartilage), 3% (OA meniscal cartilage), and 26% (OA articular cartilage) of the rate in dentin. Compared with Leu in dentin, joint tissue Leu racemized at 168% (normal articular cartilage), -43% (OA meniscal cartilage), and 97% (OA articular cartilage) of the rate in dentin. The age-related racemization (linear regression slope) of OA articular cartilage and OA meniscal cartilage extracellular matrix proteins (D/D+L ratio by age) was also compared with the normal articular cartilage using linear regression slopes. Compared with Asx in normal articular cartilage, joint tissue racemized at 23% (OA meniscal cartilage) and 46% (OA articular cartilage) of the rate in normal articular cartilage (Figure 2). Compared with Ser in normal articular cartilage, joint tissue racemized at 5% (OA meniscal cartilage) and 38% (OA articular cartilage) of the rate in normal articular cartilage. Compared with Leu in normal articular cartilage, joint tissue racemized at -26% (OA meniscal cartilage) and 52% (OA articular cartilage) of the rate in normal articular cartilage.

**Figure 3:**
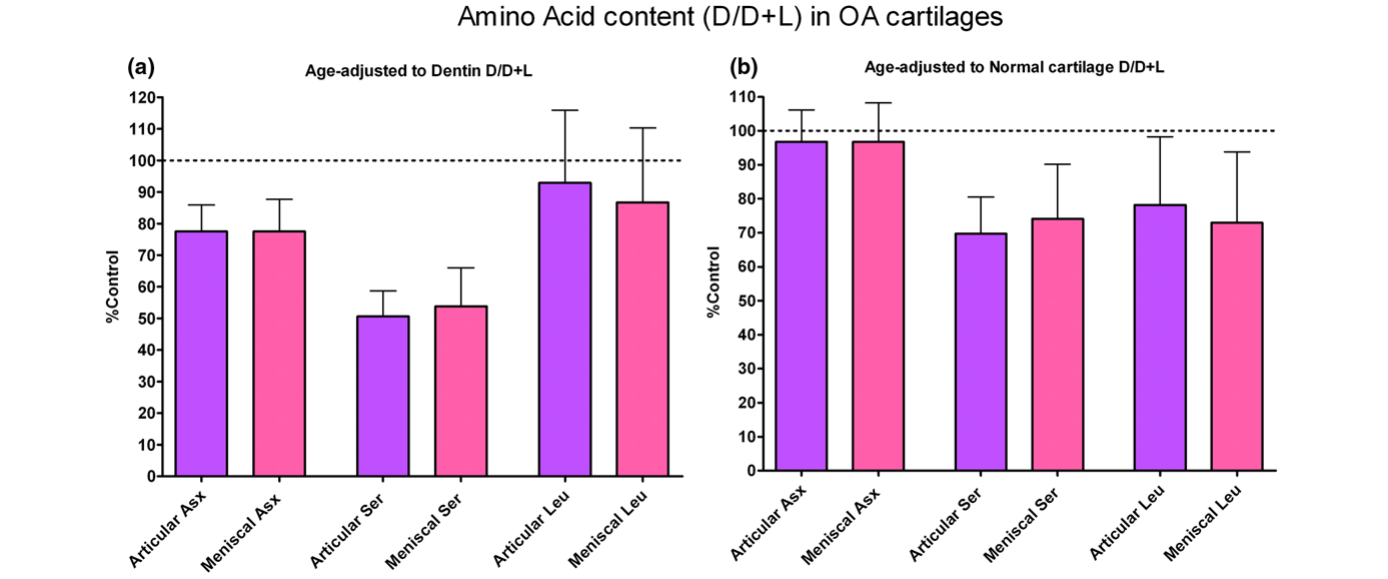
Age-adjusted D/D+L expressed as a percentage of the dentin control material and the normal articular cartilage control material. The paired *t *test comparing osteoarthritic (OA) articular cartilage with OA meniscal cartilage was not significant. The analysis of variance result for asparagine + aspartate (Asx), serine (Ser), and leucine (Leu) was *P *< 0.0001. The Bonferroni post test showed significant differences (*P *< 0.001) between all three amino acids (Asx, Ser, and Leu). **(a) **Dentin as control (mean percentage of control + standard deviation). **(b) **Normal articular cartilage as control (mean percentage of control + standard deviation). Purple represents OA articular cartilage, and pink represents OA meniscal cartilage.

Age-adjusted D/D+L ratios for OA articular cartilage and OA meniscal cartilage were derived using the regression equations of the lines for the amino acid in the dentin control material and the normal articular cartilage control material as standard curves (Figure 2). Specifically, age (x) of an articular cartilage or meniscal cartilage sample was input into the equation (y = a + bx) to generate a theoretical maximal D/D+L value (y) for a non-metabolizing tissue (dentin) and a normally metabolizing tissue (normal articular cartilage). Measured D/D+L was then divided by this theoretical control value to give an age-adjusted percentage of control. The age-adjusted proportions of racemized amino acids (D/D+L expressed as a percentage of the control material) were compared using paired *t *analysis between OA articular cartilage and matched OA meniscal cartilage (Figure 3). An age-adjusted D/D+L ratio of 100% would indicate a maximal rate of racemization for that amino acid when compared with dentin and, in our interpretation, would represent no tissue turnover. An age-adjusted D/D+L ratio of 100% when compared with normal articular cartilage would represent normal tissue turnover. Values of less than 100% are taken as an indicator of increased amino acid turnover relative to the control. Whether adjusted to dentin or normal articular cartilage, the age-adjusted D/D+L ratios in OA articular cartilage were similar (*P *> 0.05) to those of OA meniscal cartilage; however, these ratios differed significantly by amino acid when compared by ANOVA (*P *< 0.0001). Bonferroni post test showed significant differences (*P *< 0.001) between all three amino acids (Asx, Ser, and Leu), demonstrating accelerated turnover for Ser and Leu in OA cartilages compared with normal cartilage.

## Discussion

We found significant age-dependent accumulation of D amino acids (slight increase in D/D+L ratio) in a metabolically inactive control material (dentin) as well as in a control material with normal metabolism (normal articular cartilage) to be Asx > Ser > Leu, with no age-dependent accumulation seen for Ile, Glx, or Ala, even though measurable amounts of the D and L forms of all six amino acids were present in all samples. We therefore could not confirm previous reports of measurable age-related racemization of all six amino acids during the timescale of human biological aging [13,14]. The presence of readily measurable amounts of these amino acids in dentin and normal articular cartilage rules out scarcity of a particular amino acid in these tissues as a confounding factor. The strong correlations in dentin of Asx (*r*^2 ^= 0.961) and Ser (*r*^2 ^= 0.8266) with age were in agreement with a previous report [15] and verify the utility of our method for measuring protein aging and turnover in biological materials.

The significant age-related differential accumulation of D amino acids (slight increase in〈 D/D+L ratio) we observed in normal articular cartilage (Asx > Ser > Leu) was somewhat similar to other reports in that Asx was greater than Ser or Leu [13,14]. For instance, in one previous report, the differential rates of accumulation of the D forms in proteins, as measured by the D/D+L ratio, were previously reported as Asx > Glx > Ser > Ala > other amino acids (tyrosine and histidine) [13]. For bone, artificially aged using either elevated temperatures or fossil bones, the rates were found to be Asp > Ala ≈ Glu > Leu ≃ Ile [14]. The negative rate of racemization we observed for Leu in OA meniscal cartilage when compared with dentin or normal articular cartilage is likely due to the much weaker correlation of this particular amino acid with age. While still significant, the correlation of Leu with age was much weaker than for Asx or Ser.

The age-related accumulation of D-Asx we observed in normal articular cartilage (D/D+L versus age, *r*^2 ^= 0.778) is consistent with previous reports [2,3,10] describing a strong association of D-Asp with age in normal articular cartilage (*r*^2 ^= 0.903) and normal rib cartilage (*r*^2 ^= 0.58 to 0.94) but stands in contrast to a recent report [16] showing very little association with age (*r*^2 ^= 0.123) in normal articular cartilage. The results of this last study are perhaps not surprising given the fact that it evaluated only D-Asp versus age, instead of the ratio of either D/L or D/D+L, which are the accepted methods of comparison in the literature. The age-related rate of racemization (slope of D/D+L versus age) we observed for Asx from normal articular cartilage was 35% higher than previously reported for healthy unaffected articular cartilage [2]. This difference is possibly due to differences in the relative health of the cartilages used for the studies. Our normal articular cartilages were derived from non-arthritic joints at the time of autopsy or surgical repair for acute trauma. Another possible contributor to this difference is the hydrolysis procedure, which underscores the need for including control material within a study of this type as well as the difficulty in comparing racemization rates between studies. Interestingly, in our study, the total amino acid content (per milligram of tissue) of apparently undamaged OA articular cartilage was 35% to 38% lower than that of normal age-matched articular cartilage. This lower amino acid concentration in normal-appearing articular cartilage from an OA joint could be due in part to increased water content from tissue swelling coincident with a loss of collagen network integrity in early OA. This is consistent with the increased water capacity of proteoglycan when the collagen network is disrupted [17]. When considered along with the lack of correlation with age for D/D+L in OA cartilages, which manifests as a 48% to 62% decrease in amino acid racemization rates when compared with normal cartilage, this is compatible with known accelerated turnover in seemingly normal regions of OA articular cartilage [18] and points to the significant changes that occur in OA cartilage before damage becomes visually apparent.

Of great significance was the finding that the age-adjusted proportions of the D amino acids, Asx, Ser, and Leu, differed by amino acid type. There are several possible interpretations of these findings. For one, these various amino acids may reflect different protein pools in articular and meniscal cartilages turning over at different rates. For instance, since there are six times as many Ser residues in proteoglycan compared with collagen II, the larger increase in Ser turnover could indicate greatest turnover of this pool. This is consistent with a previous report that showed a 60% decrease in the D/L Asp ratio in cartilage samples that were enzymatically depleted of proteoglycan [19] and with a more detailed study that showed that the majority of the D-Asx accumulation in normal articular cartilage occurred in the hyaluronan-binding domain of the A1D1 fraction of proteoglycan [20]. Different age-adjusted proportions of the D amino acids may also vary from one another as a result of hot spots for racemization affecting a particular amino acid differentially.

In contrast to dentin, articular and meniscal cartilages undergo protein turnover, so the apparent rates of racemization in these tissues represent the net accumulation due to age, counteracted by the combination of anabolic and catabolic processes. In normal and non-lesioned OA articular cartilage, there was no protein loss with age. Without the availability of racemization data, one might infer that this tissue was therefore inert. However, in light of the difference in racemization rates relative to the standard, it is clear that non-lesioned OA articular cartilage evinced increased anabolism balanced by catabolism (Table 3). In contrast, the amino acid content of meniscal cartilage diminished with age, while racemization rates were similar to those of OA articular cartilage. This is compatible with an imbalance of protein catabolic and anabolic processes with catabolism exceeding anabolism in meniscal cartilage with the residual mass made up of non-proteinaceous material (for example, glycosaminoglycan or water due to swelling). Thus, the availability of racemization data allows inferences regarding tissue turnover, and in particular the state of anabolism, that would otherwise be unavailable through traditional biochemical methods. This finding that OA meniscal cartilage protein loss exceeds that of OA articular cartilage adds to the increasing evidence that early pathological changes in meniscus are important to the development of OA [21].

**Table 3:** Expected changes with age in total amino acid content (D+L) and amino acid racemization (D/D+L)

	Total amino acid (D+L) with age	Ratio D/D+L with age
Anabolism	Slight increase	Slight decrease
Catabolism	No change	Slight increase
Anabolism = Catabolism	No change	Slight decrease
Theoretical normal cartilage aging	No change	Moderate increase
Swelling and/or glycosaminoglycan increase	Slight decrease	No change
Normal articular cartilage results	No change	Moderate increase
Osteoarthritic articular cartilage results	No change	No change
Osteoarthritic meniscal cartilage results	Slight decrease	No change
Dentin results	No change	Large increase

## Conclusions

In summary, analyses of the D amino acid content of joint tissues provided valuable insights into their potential for anabolism or repair, demonstrating comparable anabolic responses for non-lesioned OA articular and meniscal cartilages. The novel determination of age-adjusted proportions of D amino acids revealed evidence for variation in the relative turnover of specific amino acids within joint tissues. Whereas some other studies have corrected for the background racemization inherent in the sample preparation, this is the first study to use an entire range of age-matched control material to adjust for this background and provide the means to accurately determine protein turnover. This method provides a new means for exploring tissue anabolism and racemization hot spots in different proteins and protein pools within a tissue.

## Abbreviations

Ala: alanine; ANOVA: analysis of variance; Asn: asparagine; Asp: aspartate; Asx: asparagine + aspartate; Gln: glutamine; Glu: glutamate; Glx: glutamate + glutamine; HPLC: high-performance liquid chromatography; Ile: isoleucine; Leu: leucine; OA: osteoarthritic; Ser: serine.

## Competing interests

The authors are applying for a patent related to the content of this manuscript.

## Authors' contributions

TVS carried out all of the laboratory analyses and drafted the manuscript. SSB advised on and coordinated the collection of teeth. RDZ participated in study design for collecting normal cartilage and in manuscript editing. VBK conceived of the study, participated in its design and coordination, and helped to draft the manuscript. All authors read and approved the final manuscript.
